# Development of a high-throughput flexible quantitative suspension array assay for IgG against multiple *Plasmodium falciparum* antigens

**DOI:** 10.1186/s12936-018-2365-7

**Published:** 2018-05-29

**Authors:** Itziar Ubillos, Joseph J. Campo, Alfons Jiménez, Carlota Dobaño

**Affiliations:** 1 ISGlobal, Hospital Clínic–Universitat de Barcelona, Barcelona, Catalonia Spain; 2 Antigen Discovery, Inc, Irvine, CA USA; 3 CIBER Epidemiología y Salud Pública (CIBERESP), Barcelona, Spain

**Keywords:** Quantitative suspension array technology, Multiplex, IgG, *Plasmodium falciparum*, Assay conditions, Assay performance, Precision, Reproducibility

## Abstract

**Background:**

Antibody responses to *Plasmodium falciparum* play a critical role in disease control. Finding reliable IgG biomarkers of protection is complicated by a parasite proteome of over 5000 proteins, some with polymorphisms. Studies of anti-malarial naturally acquired and vaccine immunity would benefit from a standard high-throughput immunoassay to measure multiple antibodies. A multiplex quantitative suspension assay to measure antigen-specific IgGs was developed and its precision (reproducibility and repeatability), dynamic range, limits of detection and quantification, and non-specific binding to different *P. falciparum* proteins tested. A set of 288 human plasma samples from a malaria-endemic region were analysed twice by two different operators. Another set of samples from 9 malaria-naïve and 10 malaria-exposed individuals were repetitively assayed during 22 consecutive days. Positive controls, negative controls, blanks and microspheres coated with bovine serum albumin were included in all assays.

**Results:**

The multiplex quantitative suspension assay demonstrated low non-specific signal and good estimates of precision and reproducibility between operators. The overall mean of non-specific binding measured in 288 plasma samples was 32.83 to ± 44.81 median fluorescence intensity (MFI). Repeatability was 7.66% ± 15.89 between triplicates for all antigens and samples, being lower in samples from malaria-exposed than malaria-naïve individuals. No evidence of significantly different variance across days in MFI or arbitrary units (AU)/mL was found, assuming homogeneity of variance between days of analysis. Intra-class correlation coefficient between 22 days of analysis was 0.98 (0.97–0.98) for MFI units and 0.9 (0.87–0.93) for AU/mL. Reproducibility between operators for all samples and antigens had an overall adjusted correlation of 0.929 for MFI and 0.836 for AU/mL.

**Conclusions:**

This high-throughput multiplex immunoassay is simple and highly reproducible. This represents an asset for malaria vaccine studies involving CSP-specific antibodies and selected antigens for sero-epidemiological purposes. Measuring a multiplex antigen panel in a single reaction will help to assess not only vaccine immunogenicity but also potential malaria vaccine effects on naturally acquired immune responses. This will accelerate the identification of immune correlates of protection, down-selection of vaccine formulations, antigen discovery and guide second-generation vaccine design.

**Electronic supplementary material:**

The online version of this article (10.1186/s12936-018-2365-7) contains supplementary material, which is available to authorized users.

## Background

Antibody responses to *Plasmodium falciparum* parasites play a critical role in disease control [1]. Finding reliable biomarkers associated with malaria protection or risk is complicated by a parasite with a proteome of over 5000 proteins (http://www.plasmodb.org), many of them differently expressed during distinct life-cycle stages and presenting genetic polymorphisms [2]. Antibodies can persist in blood for months after infection [3] and therefore have been used as indicators of malaria transmission [4–7]. In a low endemic setting, the use of direct parasitological methods such as microscopy, rapid diagnostic tests (RDTs) or quantitative polymerase chain reaction (qPCR) lack sensitivity because of low numbers of positive samples [8]. Also, direct parasitological detection methods only provide a snapshot of the present malaria infection, whereas serological markers can better capture past (as well as present) malaria exposure and reflect malaria transmission over a prolonged period of time [9]. A high-throughput assay for the measurement of multiple antigen-specific immunoglobulins (Ig) would be very useful for studies of malaria naturally acquired or vaccine-induced immunity, as well as other sero-epidemiological biomarker studies.

Quantitative suspension array technology (qSAT) is a high-throughput immunoassay that has advanced biomarker research compared to traditional methods, such as ELISA, and has been employed for measurement of DNA, cytokines and antibodies [10]. When used in multiplex format, qSAT dramatically reduces labour input, reagents and sample volume requirements [11]. Quantitative measurements are as important in immunological investigations as qualitative determinations of seropositivity or seronegativity, where definitive thresholds may be identified. Inclusion of calibration curves using standard reference samples allows for quantitative or semi-quantitative estimates, either in physiological concentration (e.g., μg/mL) or arbitrary units (e.g., AU/mL). The problem with many standardized assays is the use of arbitrary measurements, such as ELISA units/mL (EU/mL), which often make results incomparable with other studies or across laboratories. A standardized assay would be more useful if it had easily interpretable measurements that could be replicated in other laboratories or with different protocols. One challenge of multiplex technology is the increasing difficulty of procuring species-specific reference standards that can be employed in a multiplex panel to produce calibration curves for all targets in the panel. Another intrinsic problem of ELISA or qSAT is that human sera may contain up to 40% heterophilic antibodies that non-specifically bind to different antigen-coupled beads, resulting in some levels of background [12, 13] that need to be evaluated.

Precision and accuracy are common metrics for evaluation of new methodologies, as well as establishing the assay’s limits of detection and quantification. The precision of an analytical procedure expresses the closeness of agreement (degree of scatter) between a series of measurements obtained from multiple sampling of a homogeneous sample under prescribed conditions. The precision of an analytical procedure is usually expressed as the variance, standard deviation (SD) or coefficient of variation (CV) of a series of measurements. Precision may be considered at three levels: repeatability, intermediate precision and reproducibility. *Repeatability* refers to a precision estimate obtained from replicate measurements made in one laboratory by a single operator using the same equipment over a short time scale. It is typically used to estimate the likely difference between replicate measurements obtained in a single batch of analysis. Therefore, repeatability is also referred to as intra-assay precision [14]. *Intermediate precision* refers to a precision estimate obtained from replicate measurements made in a single laboratory under more variable conditions than repeatability conditions. Intermediate precision is also known as ‘between-laboratory reproducibility’ if tested in different laboratories or ‘between-day reproducibility’ if tested in different days [14, 15]. *Reproducibility* commonly refers to a precision estimate obtained from replicate measurements carried out with different laboratories, operators and equipment. It is also known as ‘inter-laboratory reproducibility’ [14]. Accuracy refers to the closeness of agreement between a measurement and the true value, and therefore includes the effect of both precision and bias [14, 16]. It encompasses both systematic and random error components. Accuracy of a method can be estimated by using certified reference materials by the new method, or by comparing the results of a new method with the results of a reference method.

The high-throughput qSAT IgG assay described in this study could be used as a standard immune-assay for malaria immunology and vaccine studies of multiple antibody targets, and to characterize antibody reactivities for sero-epidemiological purposes. Here, a qSAT IgG assay with a set of malaria antigens was developed and the dynamic range and the limits of detection and quantification assessed. The analysis of the assay for nonspecific binding and precision (reproducibility and repeatability) is reported.

## Methods

### Study samples

Plasma samples used in the establishment of the qSAT assay were obtained from children and adult volunteers naturally exposed to malaria, participating in two studies conducted at the *Centro de Investigação em Saúde da Manhiça* (CISM), southern Mozambique. One set of samples were from a clinical trial of intermittent preventive treatment during pregnancy with sulfadoxine-pyrimethamine (IPTp-SP) (Clinicaltrials.gov identifier NCT00209781) conducted in 2003–2005 [17]. A unique operator in 22 consecutive days and in triplicates assayed plasmas from this study by qSAT to assess precision of the assay. Another set of samples were from a clinical trial of different chemoprophylaxis schedules to selectively control first exposure to *P. falciparum* in infancy (Clinicaltrials.gov identifier NCT00231452) conducted in 2005–2009 [18]. Two operators assayed samples from this second study in 5 different days. A pool of hyperimmune plasmas (HIP) from Mozambican adults life-long exposed to malaria was used as a polyclonal positive control and antigen-specific reference standard [19]. Negative controls from malaria-naïve volunteers were obtained from ISGlobal repository. The analysis of all the samples was covered under protocols approved by the National Mozambican Ethics Review Committee and the Hospital Clínic of Barcelona Ethics Review Committee, and written informed consent was obtained from all participants or their parents/guardians before collection of specimens.

### Antigens and antibodies

A combination of 11 antigens expressed during the pre-erythrocytic and erythrocytic stages of *P. falciparum* life cycle was selected for the qSAT multiplex panel. The apical membrane antigen (AMA)-1 of the 3D7 parasite strain [20–22], the F-2 region of the erythrocyte binding antigen (EBA)-175 [22, 23], the Duffy binding-like (DBL)3x domains and DBL-α of the erythrocyte membrane protein (PfEMP)-1 were produced at ICGEB (Delhi, India) [24, 25]. The AMA-1 and the 42 kDa fragment of the merozoite surface protein 1 (MSP-1_42_) from the FVO strain were provided by WRAIR (Walter Reed Army Institute of Research, MD, USA) [26–28]. The liver stage antigen (LSA)-1 [29, 30], the sporozoite surface protein 2 (SSP2 or TRAP) [31], the circumsporozoite protein (CSP) [32] and the cell traversal-ookinete surface antigen (CelTOS) [33] were purchased from Protein Potential, LLC (Rockville, MD, USA).

Sp3C6 monoclonal antibody (mAb) produced in mice was gifted to this study by the Pluschke lab at the Swiss TPH Institute (Basel, Switzerland). Sp3C6 mAb gives specific responses to CSP of the 3D7 *P. falciparum* strain [34].

### Microsphere coupling

A qSAT multiplex panel was constructed to quantify IgG responses to *P. falciparum* antigens using Luminex xMAP™ technology (Luminex Corp., Austin, TX, USA) and the Bio-Plex 100 platform (Bio-Rad, Hercules, CA, USA). MagPlex polystyrene 6.5 μm COOH-microspheres (Luminex Corp, Austin, TX, USA) of different ID regions were selected for each antigen, including one for bovine serum albumin (BSA). For the standard curve, microspheres were coupled to anti-human IgG F’ab antibody (Sigma-Aldrich, Madrid, Spain). For comparison of the anti-IgG standard curve with a curve generated from the anti-CSP Sp3C6 mAb, microspheres were coupled to anti-mouse IgG F’ab antibody (Jackson ImmunoResearch Inc. PA, USA). Briefly, microspheres were washed, sonicated and activated with Sulfo-NHS (*N*-hydroxysulfosuccinimide) and EDC (1-Ethyl-3-[3-dimethylaminopropyl]carbodiimide hydrochloride) (Pierce, Thermo Fisher Scientific Inc., Rockford, IL, USA). Microspheres were washed and resuspended in cold Dulbecco’s PBS (dPBS) pH 7.2 (Invitrogen, Carlsbad, CA, USA) or 50 mM MES pH 5.0 (Sigma, Tres Cantos, Spain), depending on the optimal buffering system for each individual antigen. The recombinant proteins were added to the tubes in concentrations ranging from 30 to 50 μg/mL and left at 4°C on a shaker overnight. Coupled microspheres were resuspended in PBS with 1% BSA and 0.05% sodium azide (PBS-BN) to block. Microspheres recovery was quantified on a Guava PCA desktop cytometer (Guava, Hayward, CA, USA). Equal amounts of each antigen-coupled microspheres were combined in multiplex tubes and stored at 1000 microspheres/μL at 4°C, protected from light. Anti-IgG-coupled microspheres were stored at 2000 microspheres/μL at 4°C, protected from light in singleplex. Bead blocking agent BSA in the coupling buffers to covalently ‘block’ the free carboxylic group (-COOH) from the microspheres was included, absorbing most of the non-specific binding to secondary or tertiary antibodies during assay steps [12] and heterophilic antibody binding seen in previous systems [13]. Also a BSA-coated microsphere in the multiplex panel was included, to determine non-specific ‘bead binders’ of serum IgG to the BSA.

### qSAT assay

Antigen-coupled microspheres were added to a 96-well μClear^®^ flat bottom plate (Greiner Bio-One, Frickenhausen, Germany) in multiplex (1000 microspheres per analyte per well) in a volume of 50 μL of Luminex Buffer (PBS-BN). Anti-IgG-coupled microspheres were added to the plate in singleplex (2000 microspheres per analyte per well) in 50 μL of Luminex Buffer. 50 µL of test plasma samples diluted 1:250 and 1:10,000 in Luminex Buffer were added to the plates in duplicates (final dilutions of 1:500 and 1:20,000, respectively). The HIP pool was used as a positive control and included on each assay plate diluted 1:150,000. Technical blanks consisting of Luminex Buffer and microspheres without samples were added in duplicate wells to detect and adjust for non-specific microsphere signal. Plates were incubated for 1 h at room temperature in agitation and protected from light. Then, washed three times with 100 μL PBS-T (0.05% Tween 20 in PBS) on a Bio-Plex Pro wash station with magnetic platform (Bio-Rad, Hercules, CA, USA). 100 μL of biotinylated anti-human IgG (Sigma-Aldrich, Tres Cantos, Spain) diluted 1:2500 in Luminex buffer was applied to all wells and incubated for 45 min as before. For the assay of anti-CSP Sp3C6 mAb and mouse IgG standard curves, biotinylated anti-mouse IgG (Sigma-Aldrich, Madrid, Spain) was used. After washing plates, 100 μL of streptavidin-conjugated R-phycoerythrin (Invitrogen, Carlsbad, CA, USA) diluted 1:1000 (1 μg/mL) in Luminex Buffer was applied to all wells and incubated for 25 min as before. Plates were washed and microspheres resuspended with 100 μL of Luminex Buffer, and covered with an adhesive film and stored at 4°C overnight to be read the next morning. Data were acquired on a Bio-Plex 100 reader using Bio-Plex Manager version 4.0 (Bio-Rad, Hercules, CA, USA). At least 50 microspheres per analyte were acquired, and median fluorescence intensity (MFI) was reported for each analyte.

### Standard curve

A heterologous reference standard [35–37] for estimating concentration of antibodies in plasma was constructed using microspheres coupled to anti-human IgG F’ab region and dilution series of IgG purified from human serum (Sigma-Aldrich, Tres Cantos, Spain). The commercially available purified human IgG was incubated with the anti-human IgG F’ab microspheres in a 10-step dilution series (twofold) starting at 250 ng/mL and producing an 11-point curve. For comparison of a heterologous standard with a homologous standard, a curve was generated with purified mouse IgG (ThermoFisher, Spain) and microspheres coupled with anti-mouse IgG F’ab region (Jackson ImmunoResearch Inc. PA, USA) plus biotinylated anti-mouse IgG (Sigma-Aldrich, Madrid, Spain). This curve was contrasted with a curve generated with a dilution series of anti-CSP Sp3C6 mAb with CSP-coupled microspheres in singleplex. The 5-PL regression was the selected method for fitting curves due to its superior fit to antibody data:

$$y = A + \frac{D}{{\left( {1 + \left( {{\raise0.7ex\hbox{$x$} \!\mathord{\left/ {\vphantom {x C}}\right.\kern-0pt} \!\lower0.7ex\hbox{$C$}}} \right)^{B} } \right)^{G} }}$$

where A is the lower asymptote (Emin), B is the slope at the inflection point (Hill), C is the concentration at the inflection point (EC50), D is the upper asymptote (Emax), and G is a factor of asymmetry added in the 5-PL regression model [38]. If the 5-PL regression model did not converge, then, a 4-PL method without asymmetry factor G was fitted instead. To obtain AU/mL, corresponding MFI values were adjusted by their corresponding blank values before curve fitting.

An *r*^2^ cut-off value of 0.994 was used for each standard curve as acceptability criteria. Additionally, blank values of all antigen-coated microspheres had to be below 200 MFI, and for the anti-IgG-coated microspheres had to be below 300 MFI. The parameters of the anti-IgG standard curve were used in a Microsoft Excel template to calculate antigen-specific AU/mL, respectively. Using the derived parameters of the standard curve, the estimates of concentration were multiplied by corresponding dilution factors to calculate antigen-specific AU/mL.

### Determinations of limit of blank (LOB) and limit of quantification (LOQ)

LOBs were estimated by measuring replicates of a technical blank (well without sample) and calculating the log_10_ MFI mean and the SD (LOB = mean blank + 1.645 × (SD blank)). To calculate concentration in AU/mL for each sample and antigen tested, we first adjusted each MFI value by its corresponding blank values. Then, the parameters from a non-linear 5-PL regression model obtained from the singleplex IgG standard curve were fitted in the inverse 5-PL equation for each sample and antigen. We established a ‘good range’ of quantification where percent change in AU/mL does not exceed 5% for a 1% change in MFI, and these were considered the assay LOQ. Only AU/mL measurements were adjusted by their corresponding blanks values.

### Assessment of precision

For the determination of repeatability and intermediate precision, antibody levels (in log_10_ MFI or AU/mL) were measured against BSA, AMA-1 3D7, AMA-1 FVO, MSP-1_42_ 3D7, MSP-1_19_ 3D7, EBA-175, LSA-1 and CSP in 10 malaria-exposed and 9 non-exposed individuals spanning a large range of immunogenicities. Samples were measured in triplicates on 22 different days, equivalent to 1254 measurements for the 8 antigens included. Repeatability between replicates for each antigen and day was assessed by the Intra-class Correlation Coefficient (ICC) [39], one way ANOVA [40] and CV ($\frac{SD}{Mean} \times 100$) [3, 15, 41]. Bland–Altman plots were also used to assess ‘within-day reproducibility’ [40].

To assess reproducibility, two operators performed the assay on 5 different days in the same laboratory and using the same apparatus. Operator and day effects were assessed, but inter-laboratory variation could not be assessed. IgG (in log_10_ MFI levels and AU/mL) to 11 *P. falciparum* antigens (AMA-1 3D7, AMA-1 FVO, MSP-1_42_ 3D7, MSP-1_42_ FVO, EBA-175, CelTOS, LSA-1, SSP2, DBL-α, DBL3x and CSP) and BSA were measured in 282 samples from malaria-exposed individuals, and 52 malaria-naïve individuals. Positive controls and blanks were included in all plates, and all samples were measured in duplicates.

### Assessment of accuracy

Since samples in this study had unknown antibody concentrations, standard curves with known concentrations of total IgG measured on 22 different days were used to assess accuracy and the observed and expected concentrations for each day of analysis compared. Also, as performance of total IgG measurement might not be representative of the performance of antigen-specific IgG measurements, parameters from the curve fitting of standard curves with mouse IgG were compared with anti-CSP mouse mAb Sp3C6 IgG standard curves.

### Statistical methods

All MFI or AU/mL measurements were log_10_-transformed for statistical analysis. Means and 95% confidence intervals (CI) were calculated for repeated measures. T-tests were used to assess differences between means. One-way ANOVA and Levene’s test [42] were used to assess differences between replicates. Agreement was assessed by performing Bland–Altman plots [40, 43], and reliability by the ICC from psych R package [39, 44]. SD and CVs were calculated for precision measurements. A *p* value < 0.05 was considered statistically significant. Data were analysed using R software version 3.4.1.

## Results

### Limits of blanks and limits of quantification

**Fig. 1:**
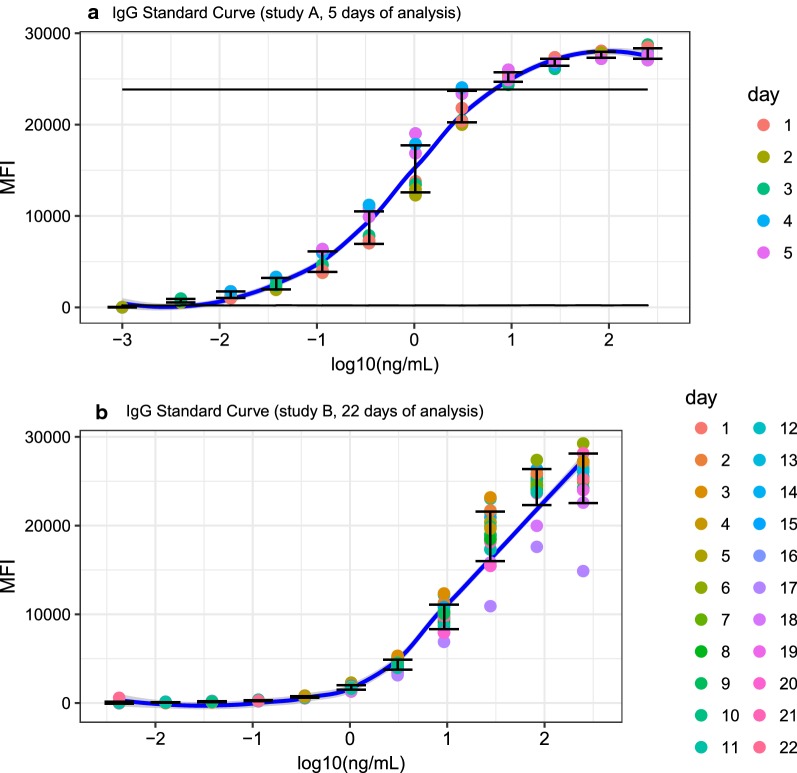
Serial dilutions of human IgG fitted into a non-linear 4-parameter logistic (4-PL) regression model. Median fluorescence intensity (MFI) values versus log_10_ IgG concentration (ng/mL). Blue line represents Loess fitted line for all days of analysis. Error bars represent the standard deviation of the mean between days of analysis. **a** Standard curves performed in 5 different days. Solid horizontal lines represent the lower limit of quantification (LLOQ) and the upper limit of quantification (ULOQ). **b** Standard curves performed on 22 different days of analysis

The LOB ranged from 95.18 MFI for the DBL-α to 150.33 for MSP-1_42_ FVO. LOB for the other antigens were: 98.43 MFI for AMA-1 3D7, 103.31 for MSP-1_42_ 3D7, 103.68 for LSA-1, 106.05 for DBL3x, 107.46 for CSP, 121.42 for CelTOS, 122.45 for AMA-1 FVO, 124.78 for SSP2, 148.44 for EBA-175 and 127.62 for BSA. The lower LOQ (LLOQ), based on an anti-IgG standard curve, was estimated at 237.88 ± 70.86 MFI, and the upper LOQ (ULOQ) at 23,355.48 ± 489.99 MFI (Fig. 1a). The analytical range was found to be 0.007–9.95 AU/mL, resulting in 2.61–233,979.78 AU/mL after correcting for the sample dilution factor (1:500 and 1:20,000) (Fig. 1b). The LLOQ (237.88 ± 70.86 MFI) was above the LOB, and measurements below the LLOQ were considered to not be quantifiable.

### Assessment of non-specific background reactivity

**Fig. 2:**
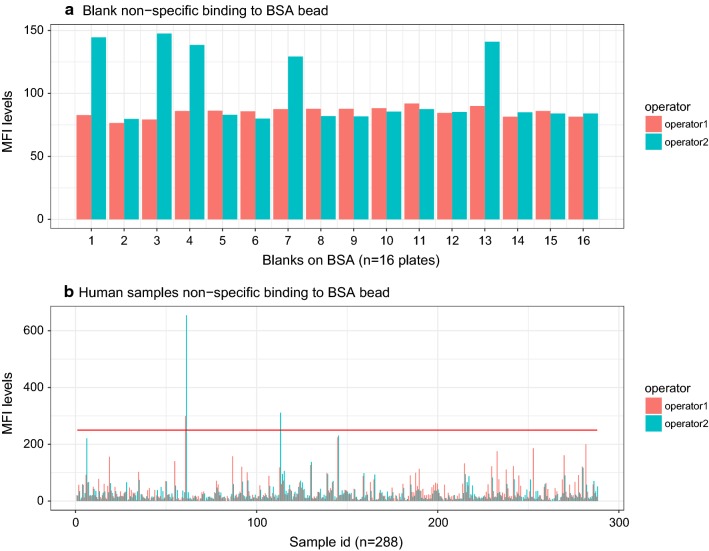
Non-specific binding in blanks and plasma samples to bovine serum albumin (BSA)-coated microspheres by operator. **a** MFI values to BSA-coated microspheres in blanks from 16 plates assayed by 2 operators; **b** MFI values to BSA-coated microspheres in 288 plasma samples, from a chemoprophylaxis clinical trial in Mozambique, assayed by 2 operators. The horizontal red line corresponds to 250 MFI. Red columns represent operator 1 and green columns operator 2

To assess non-specific background reactivity, MFI levels to BSA-coupled beads were measured in 288 samples and blanks. Signal to blanks had values below 150 MFI for both operators (Fig. 2a). The overall mean of non-specific binding to BSA in samples was 32.83 ± 44.81 MFI, with a range 0–654.5 MFI, and was different depending on the operator (Fig. 2b). In 2 of 288 samples for operator 2 (0.69%) and one for operator 1 (0.34%), the non-specific binding to BSA was above 250 MFI, one sample being the same in both operators.

### Assay precision

**Fig. 3:**
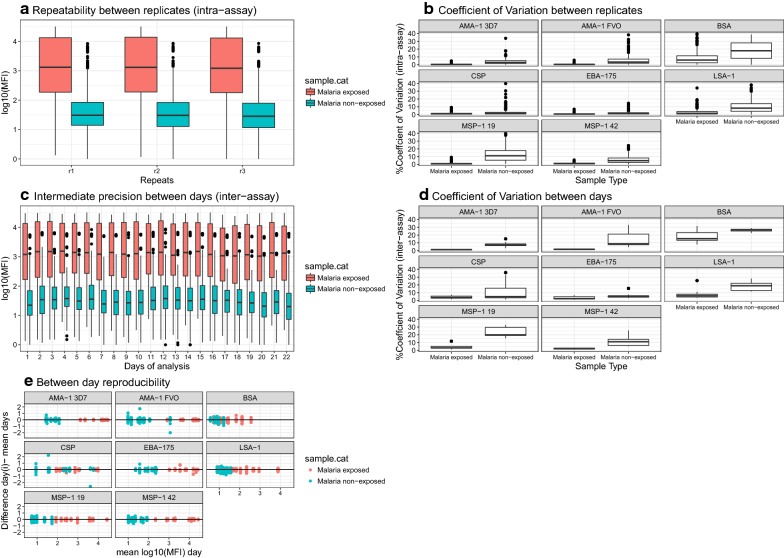
Repeatability and intermediate precision of sample measurements. IgG in samples from malaria-naïve (non-exposed) and malaria-exposed individuals from an IPTp clinical trial in Mozambique, were measured in triplicates on 22 different days against 7 *P. falciparum* antigens. **a** Repeatability represented by boxplots of log_10_ MFI distributions for all antigens together at each repetition (r1, r2, r3); **b** boxplots of coefficient of variation between replicates by antigen and sample type. **c** Intermediate precision represented by boxplots of log_10_ MFI distribution for all antigens together at each day of analysis, and **d** boxplots of coefficient of variation between days of analysis by antigen and sample type. Boxplot horizontal lines represent median and interquartile range. **e** Bland–Altman plots showing the difference of each day versus the mean of all days of analysis for each antigen and both sample types

Precision was assessed for two measurements: antibody level estimates from heterologous interpolation of an anti-human IgG standard curve (AU/mL) and log_10_ of MFI (henceforth, “MFI”). The repeatability of the assay, measured using the ICC between replicates for all antigens and days of analysis, gave a value of 1 for samples from malaria-exposed individuals (n = 1540 observations) and 0.99 for the non-exposed donors (n = 1386 observations). No evidence of significantly different variance across replicates was seen, therefore homogeneity of variance in triplicate measurements was assumed (p-value = 0.73) (Fig. 3a). Repeatability or intra-assay variability was also assessed by analysing the CV of MFI for triplicate measures [3, 16] with an overall CV of 7.66% ± 15.89 between triplicates for all antigens and samples. CV was lower in samples from malaria-exposed (2.81% ± 7.85 n = 10) than malaria-naïve (13.07% ± 20.25, n = 9) individuals (p-value < 0.001), suggesting higher variability between replicates of low immunoreactive samples. Intermediate precision or inter-assay variability was assessed by the ICC between the 22 days of analysis: 0.98 (0.97–0.98) for MFI and 0.9 (0.87–0.93) for AU/mL. Similarly, no evidence of significantly different variance across days in MFI (p-value = 0.613) or AU/mL (p-value = 0.446) measurements was shown, assuming homogeneity of variance between days of analysis. Overall, between-day or inter-assay CV was 9.24% ± 8.98 for MFI data [3, 16] and was lower in samples from malaria-exposed (5.25% ± 6.16, n = 10) than non-exposed (14.16% ± 9.44, n = 9) individuals (p-value < 0.001) (Fig. 3b). Similarly, between-day CV in AU/mL was lower in malaria-exposed individuals (31.28% ± 25.56) than malaria-naïve individuals (38.2% ± 14.78) (p-value < 0.001). To assess if variability depended on the antigen immunogenicity, mean CV was measured between replicates and days for each antigen (Table 1). BSA-coated microspheres used to control for non-specific binding gave the highest between-day variability. Between-day variability in AU/mL increased compared to that obtained with MFI, suggesting that the curve fitting did not correct the day-to-day variability. Correlations (*r*^2^) between days of analysis in MFI and AU/mL, all antigens together, were greater than 0.9 (p < 0.001) (Additional file 1). The difference in the daily MFI mean of triplicates and mean of all days was plotted against the daily mean of triplicates for each antigen and for test samples and negatives controls (Fig. 3c), which showed that variation was higher at lower MFI values.

**Table 1:** Mean coefficient of variation (CV) and standard deviation (SD)

Analyte	CV between repeats (%) log_10_ MFI (3 repeats) Intra-assay		CV between days (%) log_10_ MFI (n = 22) Inter-assay		CV between days (%) AU/mL (n = 22) Inter-assay	
	CV	SD	CV	SD	CV	SD
AMA-1 3D7	2.31	± 4.47	4.35	± 4.07	25.79	± 12.16
AMA-1 FVO	4.96	± 16.59	7.57	± 9.76	28.65	± 14.7
EBA-175	1.47	± 2.79	4.59	± 3.16	35.25	± 33.16
CSP	2.29	± 7.92	7.97	± 9.61	27.73	± 11.46
LSA-1	11.05	± 18.43	13.21	± 8.12	43.27	± 17.81
Control BSA	22.54	± 23.74	21.38	± 7.08	64.12	± 31.18
Overall	7.66	± 15.89	9.24	± 8.98	31.28	± 22.74

Between triplicates and days of analysis in log_10_MFI or AU/mL for each antigen of the multiplex panel

### Assay reproducibility

**Fig. 4:**
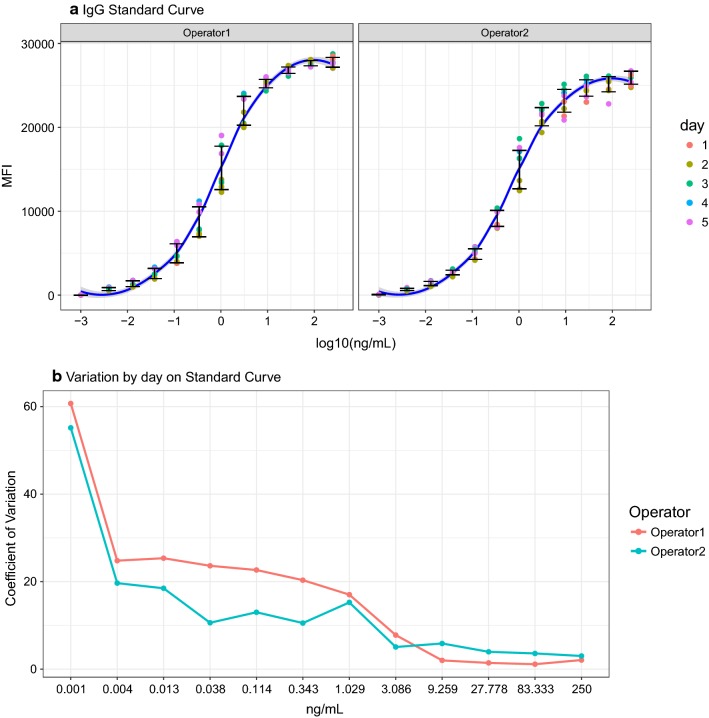
Reproducibility between days of the IgG standard curve by operator. **a** MFI versus log_10_ ng/mL by operator. Blue line represents Loess fit, and the error bars represent the standard deviation of the mean between days of analysis. **b** Between-day coefficient of variation at each dilution point and by operator

**Fig. 5:**
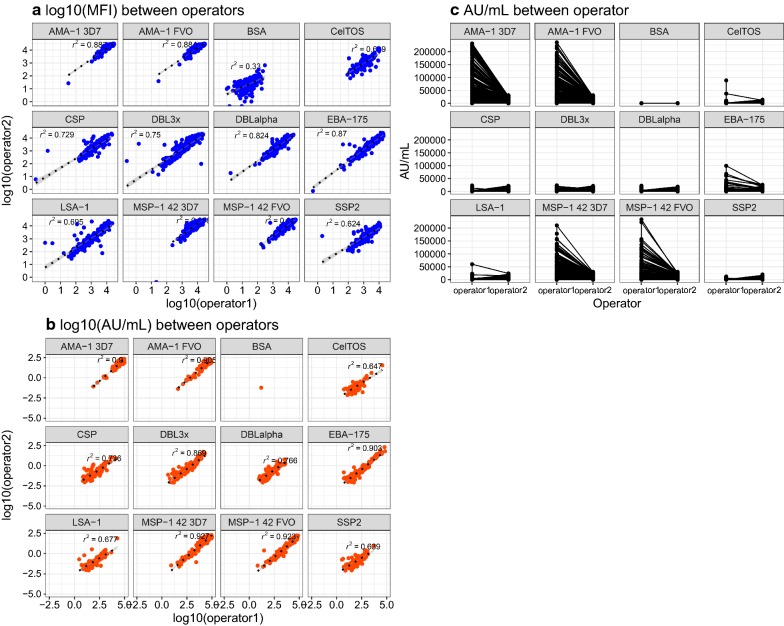
Correlation of samples measurements between operators. Plasma samples (n = 288) from a chemoprophylaxis clinical trial in Mozambique, with **a** correlation of log_10_ MFI data; **b** correlation of log_10_ AU/mL data; and **c** AU/mL at each dilution and antigen by operator. *r*^2^ is Pearson’s correlation coefficient

**Fig. 6:**
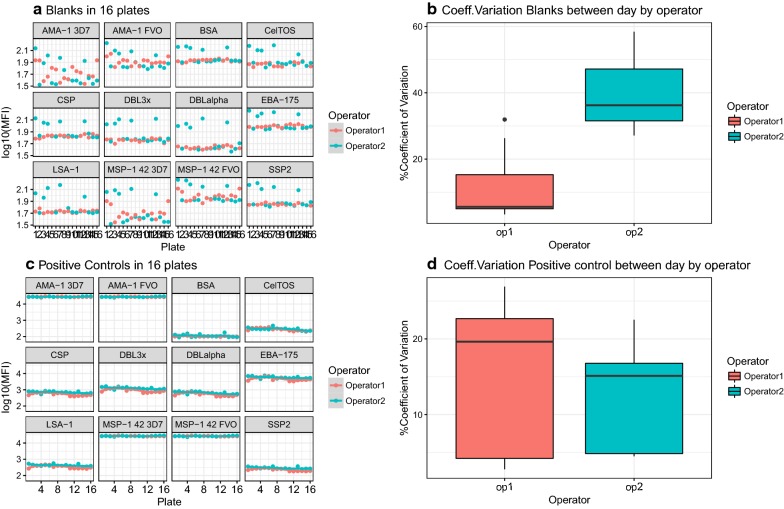
Reproducibility of blanks and positive controls. **a** log_10_ MFI levels of blanks assayed in 16 plates, and **b** boxplots of coefficient of variation of blanks in each operator between days. **c** log_10_ MFI levels of positive controls assayed in 16 plates, and **d** boxplots of coefficient of variation of positive controls in each operator between days. Boxplot horizontal lines represent the median and interquartile range

To assess the operator effect, the sample size was expanded to 288 samples against a panel of 11 *P. falciparum* antigens. The inter-assay CV of the standard curves differed between operators (p-value = 0.023), and the inter-assay variability for each operator differed depending on the dilution factor, with higher variability at lower concentrations (Fig. 4). For correlation between operators for all samples and antigens (Fig. 5), all antigens together had an overall adjusted *r*^2^ of 0.929 for MFI and *r*^2^ of 0.836 for AU/mL (log_10_ scale). Correlation of AU/mL between operators tended to increase with higher MFI, such as AMA-1, MSP-1_42_ and EBA-175 (Fig. 5a, b). Compared to MFI, the measurements in AU/mL had very different ranges (Fig. 5b, c), with higher estimates of AU/mL by operator 1 for the antigens with higher MFI; this was due to the different estimates of the parameters from the 5-PL or 4-PL regression model by operator. The between-day operator CV for blank and positive controls based on MFI, plate-to-plate signals and trend lines for each operator were not aligned for blanks (p < 0.001), although positive controls were aligned (Fig. 6).

### Assay accuracy

**Fig. 7:**
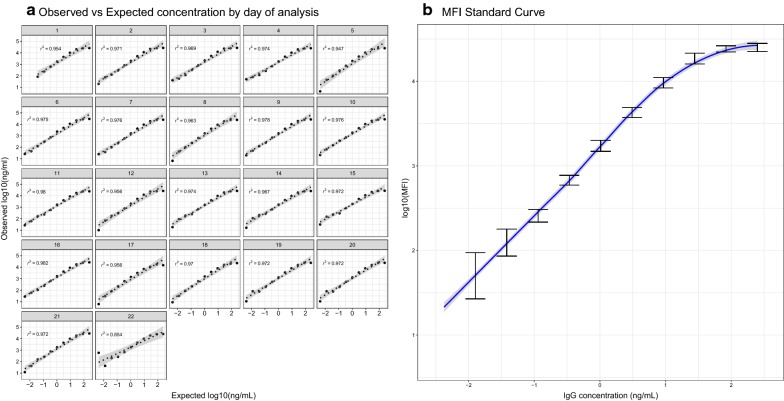
Accuracy of standard curve with human IgG assayed in 22 plates. **a** Observed versus expected concentrations after inverse non-linear 4-parameter logistic (4-PL) regression model fitting and *r*^2^ from Pearson’s correlation coefficient **b** log_10_ MFI to IgG concentration (ng/mL) median and error bar at each dilution point in the 22 plates

**Fig. 8:**
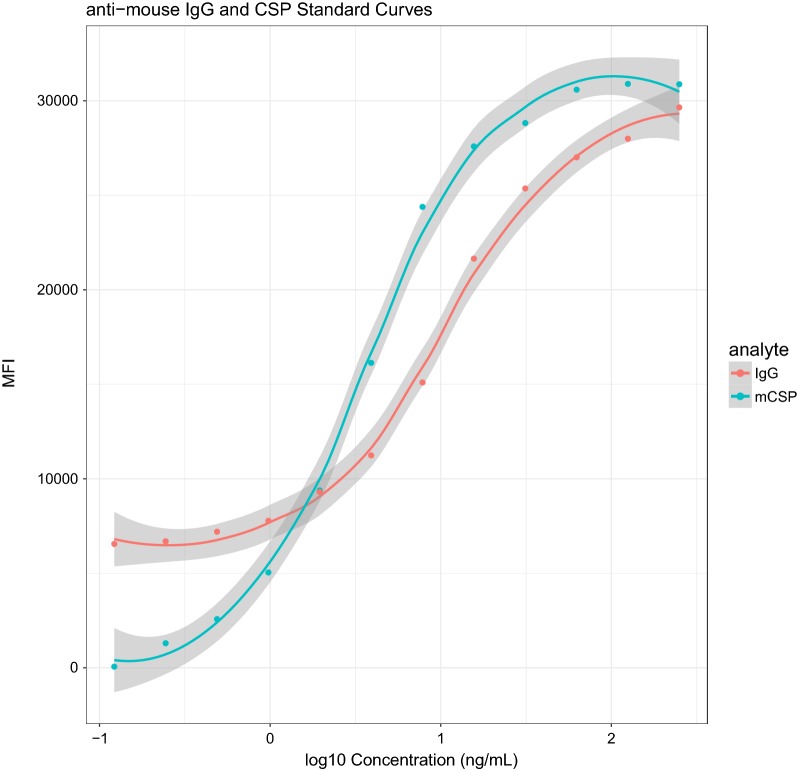
Standard curve with mouse IgG (red) and monoclonal anti-CSP IgG (blue). Lines represent Loess fitting and grey area the 95% confidence interval

Measurements of the observed versus the expected concentrations from the IgG standard curves assayed over 22 different days had a minimum *r*^2^ value of 0.854 on day 22 and all remaining days with *r*^2^ > 0.95 (Fig. 7). When a standard curve based on mouse IgG was compared to a curve based on a monoclonal mouse anti-CSP IgG, the curves showed very different estimates of the 5-PL or 4-PL regression model parameters (Table 2), particularly EC50 and minimum estimates. Measurements and concentrations of monoclonal anti-CSP IgG and purified mouse IgG were not comparable (Fig. 8).

**Table 2:** Parameters from the non-linear 4-parameter logistic (4-PL) regression function for singleplex curves

Analyte	Parameters	Coefficient	Standard error	95% CI	
IgG	Emax	30,929.13	286.52	30,280.97	31,577.28
	Emin	423.37	420.67	− 528.26	1375.00
	EC50	3.53	0.15	3.18	3.88
	Hill	1.41	0.08	1.23	1.60
mCSP	Emax	32,000.25	3650.55	23,742.15	40,258.36
	Emin	4000.20	1547.16	500.27	7500.13
	EC50	20.44	7.90	2.58	38.30
	Hill	0.87	0.27	0.26	1.48

Anti-mouse IgG-coupled beads incubated with purified mouse IgG and CSP-coupled beads incubated with mouse anti-CSP monoclonal antibody (mCSP) at known IgG concentrations

*CI* confidence interval

## Discussion

A high-throughput immunoassay such as the qSAT described herein is highly useful for biomarker research using custom antigen panels for complex pathogens, such as *P. falciparum*. It can be used as a standard immune-assay for malaria vaccine studies involving multiple antibody specificities, and to select antigens for sero-epidemiological purposes. The qSAT assay developed here demonstrated low non-specific signal, good estimates of precision and correlation between operators, albeit with operator-dependent differences in range of AU/mL estimates.

For calibration purposes, the use of a standard curve fitting an inverse 4-PL or 5-PL regression model to estimate concentration is a valid method to normalize MFI and provide quantifiable antibody measures [45], accounting for day-to-day variability. Typically, low multiplexity assays would utilize a reference sample or pool of mAb to produce standard curves for each analyte, known as ‘homologous interpolation’ [37]. Higher levels of multiplexing require standards that react strongly to all antigens in the panel or larger pools of mAb, both resources that are likely limited and not widely available. The hypothesis of the current system tested was that a single curve generated from anti-human IgG coated beads and commercially available purified IgG could close the gap for increasingly complex multiplex assays. The validity of this approach, despite the differences in binding mechanism between IgG capture and antigen-specific IgG binding, is that the secondary antibody and streptavidin-conjugated detection molecule (R-PE) are the same for all beads. Thus, the read-out of anti-hIgG and malaria antigen beads are the same: fluorescence emitted from IgG bound (or captured) to the bead. Indeed, this approach, known as ‘heterologous interpolation’ has been used previously in development of ELISA and similar assays [19–21] and is useful when standard reference reagents for homologous interpolation are unavailable. The possibility exists that systematic effects may alter the relationship of IgG captured to beads by the Fc region with that of IgG binding specifically to antigen.

For most antigens, between-day variability differed from estimated concentration in AU/mL and fluorescence-based crude MFI measurements. This variability was attributed to operator-dependent differences in the slopes and curve parameters, resulting in large deviations in AU/mL estimates. Furthermore, the comparison of the anti-IgG curve with a curve generated with a specific mAb against the CSP protein (Fig. 8) showed differing slopes and limits of the curves. These differences are possibly due to higher affinity of the mAb to the target antigen compared to the affinity of anti-IgG-coated beads. To estimate relative potency, both curves would need to have a common slope and the maximum achievable response should be identical [46]. Unfortunately, these conditions were not met for the curves generated with anti-mouse IgG and specific mouse anti-CSP mAb. Also, polyclonal biological samples like plasma or serum contain antibodies with a range of affinities, suggesting that calibration curves from either antigen-specific mAbs (homologous) or purified human IgG (heterologous) may provide a better relative estimate of antibody concentration (AU/mL) than physiological concentration (ng/mL). The standard curve with quantifiable human IgG was expected to reflect levels of IgG antibodies in serum/plasma and to be a widely applicable resource for estimating concentration. However, the poor reproducibility of AU/mL measurements using a single IgG standard curve suggests that a pool of known antibody concentrations may perform better in measuring antibodies against *P. falciparum*. In the absence of a homologous reference standard, a standardized assay with heterologous interpolation may be used.

The multiplex assays were performed with 1000 microspheres/analyte/well and the singleplex standard curve with 2000 microspheres/analyte/well. Other studies found no differences between both bead concentrations [3], although they did not measure differences when assays were performed in singleplex at 1000 microspheres/analyte/well. Here, a higher concentration of beads in singleplex assays was included for improved acquisition of the minimum number of events.

Findings from other laboratories indicate that some serum/plasma samples contain antibodies that appear to bind directly to the carboxylated surface of the microspheres even in the absence of coupled antigen [12, 13]. Here, an overall low level of non-specific binding of the samples assayed was observed by measuring response to BSA protein, with MFI generally below 150 when using blanks and with only 2 of 288 (0.69%) samples with crude MFI above 250 to BSA-coupled microspheres. Other studies have assessed non-specific binding of IgG directly to the microsphere surface by using unconjugated blank beads [47]. While useful in identifying ‘bead binding’ activity, the approach does not adequately quantify the contribution of bead-binding signal to the overall background signal of the current assay using antigen-coupled/BSA-blocked microspheres. BSA-coated microspheres better account for the signal attributable to non-specific IgG binding to (1) the microsphere surface, (2) free –COOH and (3) BSA. An added benefit is identifying outliers that represent IgG binding through specific recognition of BSA as an antigen (e.g., through various types of bovine exposure), allowing for sample censorship or adjustment of signals. Maintaining aseptic technique is critical throughout the procedure, and all batches of coupling reactions were tested with ‘blanks’ before combining into a single batch for a series of experiments. Therefore, when preparing the assay, coupling reactions of *P. falciparum* antigens with blanks above 250 MFI should be excluded from the multiplex panel.

Repeatability has traditionally been assessed by estimating the CV or relative SD [3, 16, 47, 48]. Similar to other studies, higher variability at lower MFI values [45] and comparable values of inter-assay and intra-assay variation [3, 41] were found, indicating acceptable precision of the assay. However, those measures do not take into account the multi-dimensionality of a multiplexed assay where antibodies against more than one antigen are measured at a time. ICC and ANOVA analyses can account for this dimensionality.

The qSAT assay gave highly reproducible MFI measurements, but the variation in the performance of the standard curves between operators may limit the use of AU/mL to a normalization method for single-operator studies until future assay development improves the reproducibility of IgG standard curves between operators and, presumably, laboratories. This suggests that a reference pool of known specific antibody concentrations may perform better across studies in measuring antibodies against *P. falciparum* [49]. The challenge remains in sourcing adequate serum/plasma/mAb pools that cover all antigens as panels become larger and more complex.

## Conclusion

The qSAT assay developed here demonstrated low non-specific signal, gave reproducible MFI measurements and good estimates of precision and correlation between operators. The use of a singleplex standard curve to measure antibody concentration through a non-linear parameter function added more variability to the assay. The assay with heterologous IgG standard may be used when homologous antigen-specific standards are unavailable, but should be further standardized for broad application. RTS,S and sporozoite malaria vaccine immune studies could benefit from the use of a multiplex antigen panel to measure not only vaccine-specific but also naturally-acquired immune responses and accelerate the identification of immune correlates of protection.

## Additional file

**Additional file 1.** Correlation matrix of MFI or AU/mL between days of analysis. All samples and controls are included. Upper panel report correlation coefficient (r) and p-value (p) and between days of analysis. Lower panels plot variables against each other.
