# Supplementary material for: Development of a high-throughput flexible quantitative suspension array assay for IgG against multiple Plasmodium falciparum antigens
Source: Malar J. 2018 May 29;17:216. doi: 10.1186/s12936-018-2365-7 (PMC5975539; doi:10.1186/s12936-018-2365-7)

Correlation Intra−plate 1 MFIs

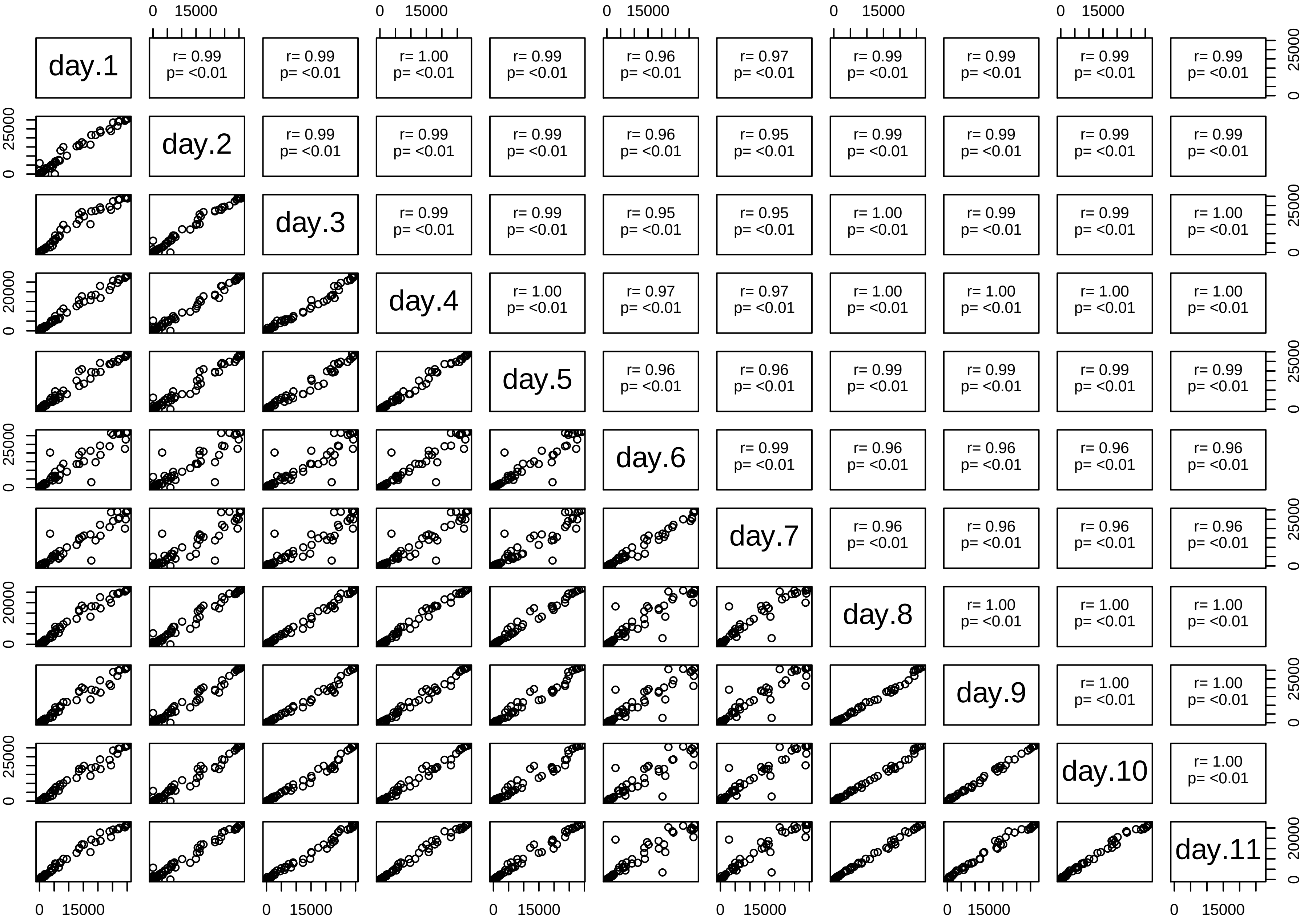

Correlation Intra−plate 2 MFIs

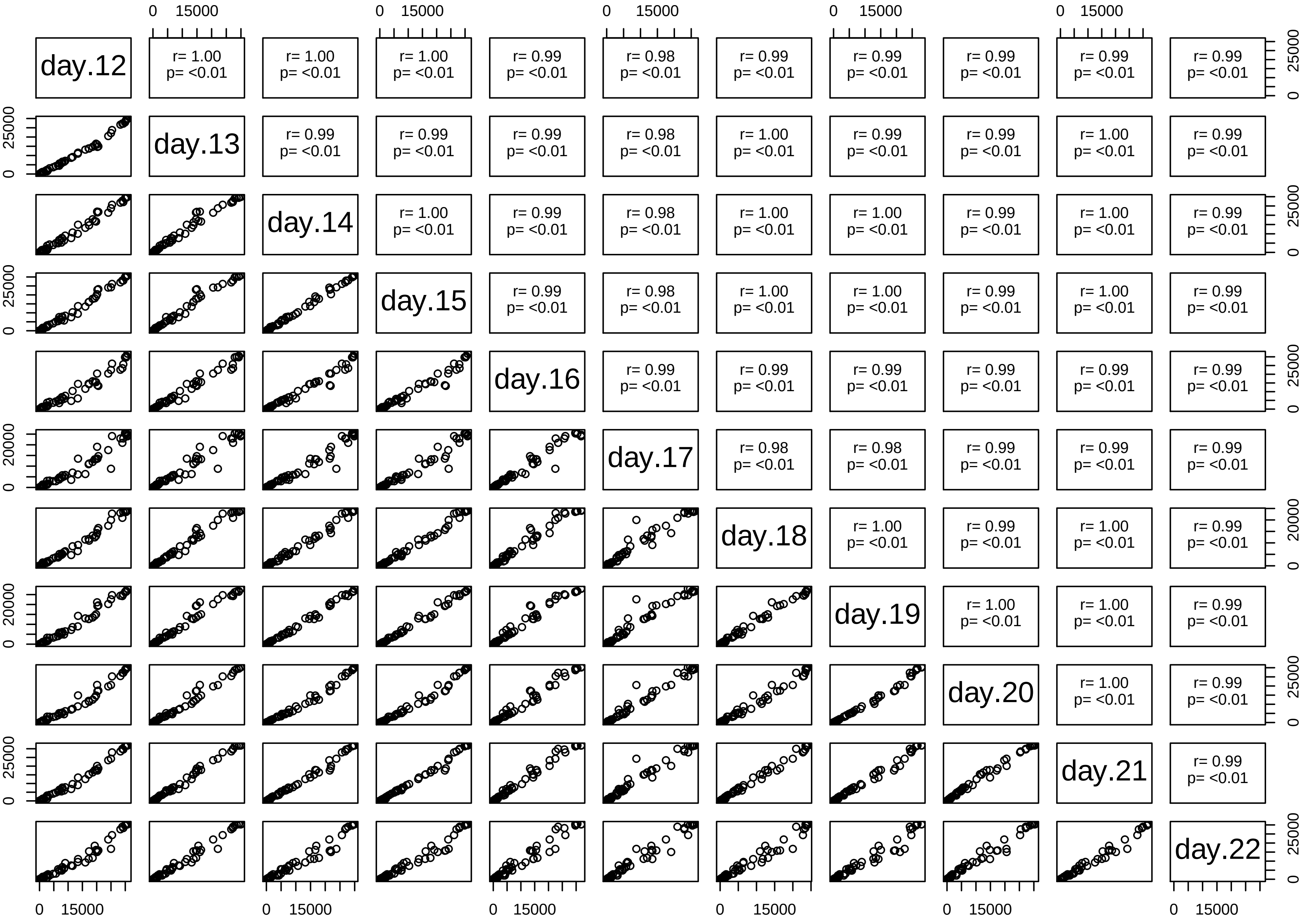

Correlation Intra−plate 1 AU/mL

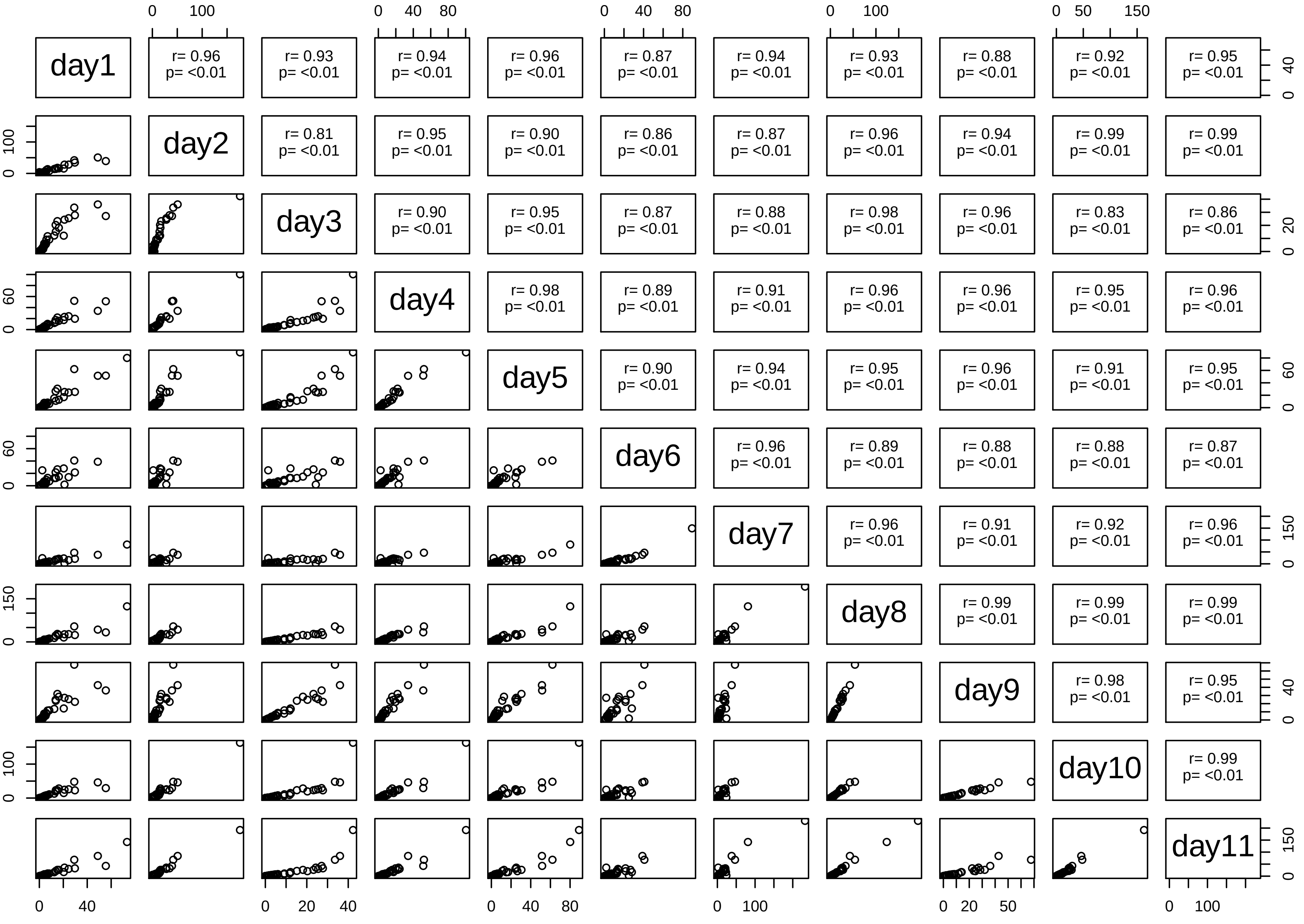

Correlation Intra−plate 2 AU/mL

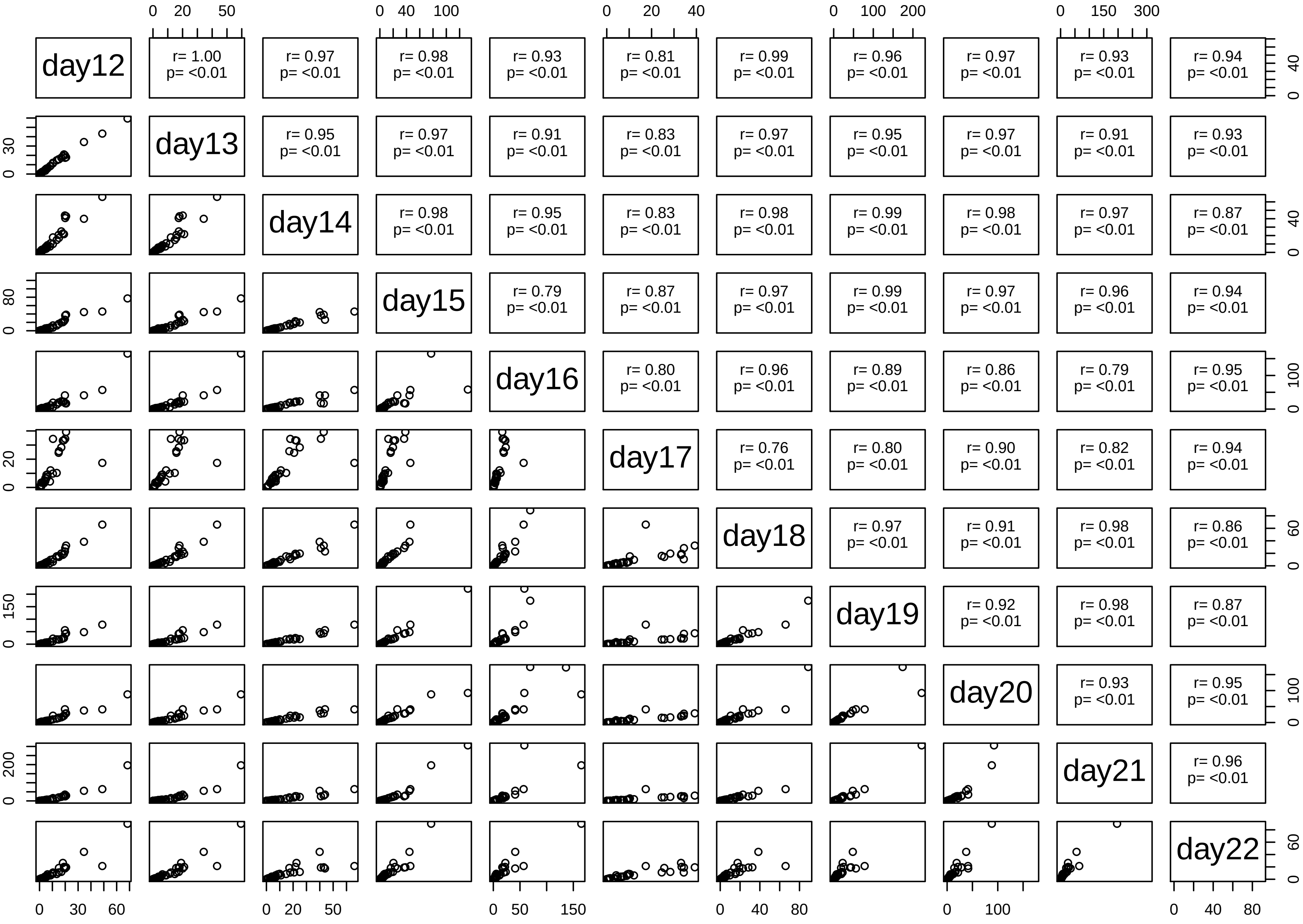

Supplement: Supplementary file 1 — Additional file 1. Correlation matrix of MFI or AU/mL between days of analysis. All samples and controls are included. Upper panel report correlation coefficient (r) and p-value (p) and between days of analysis. Lower panels plot variables against each other. [file 12936_2018_2365_MOESM1_ESM.pdf]
